# *TGIF1* overexpression promotes glioma progression and worsens patient prognosis

**DOI:** 10.1002/cam4.4822

**Published:** 2022-05-15

**Authors:** Baoya Wang, Qiong Ma, Xuelin Wang, Kunshan Guo, Zhendong Liu, Gang Li

**Affiliations:** ^1^ Department of Clinical Laboratory, Henan Provincial People's Hospital People's Hospital of Zhengzhou University, People's, Hospital of Henan University Zhengzhou People's Republic of China; ^2^ Department of Respiratory and Critical Care Medicine, Henan Provincial People's Hospital People's Hospital of Zhengzhou University, People's, Hospital of Henan University Zhengzhou People's Republic of China; ^3^ Xuchang Central Hospital of Henan University of Science and Technology Xuchang People's Republic of China; ^4^ Department of Surgery of Spine and Spinal Cord Henan Provincial People's Hospital Zhengzhou People's Republic of China

**Keywords:** biomarker, glioma, prognosis, *TGIF1*

## Abstract

Transforming growth factor β‐induced factor homeobox 1 (*TGIF1*) reportedly promotes the pathological processes of various malignant tumors. However, few studies have investigated the role of *TGIF1* in gliomas. We aimed to explore the relationship between *TGIF1* expression and the clinical characteristics of patients with glioma, including their overall survival. A total of thousands transcriptome datapoints were downloaded from public databases to determine the correlations between *TGIF1* and various clinicopathological features using the Wilcoxon or Kruskal–Wallis tests. The Kaplan–Meier and Cox statistical methods were used to explore the prognostic significance of *TGIF1*. Gene set enrichment analysis (GSEA) was used to indirectly identify the pathological mechanisms modulated by *TGIF1*, and compounds that inhibit its expression were determined using a connectivity map (CMap). *TGIF1* was significantly overexpressed in gliomas and was correlated with unfavorable prognostic factors and shorter overall survival. Cox analysis confirmed that *TGIF1* expression was a significant predictor of poor prognosis in patients with glioma. GSEA revealed that the signaling pathways associated with *TGIF1* expression in glioma included extracellular matrix receptor‐ and cell cycle‐modulating proteins. CMap analysis showed that the small molecules scriptaid, torasemide, dexpropranolol, ipratropium bromide, and harmine were potential negative regulators of *TGIF1*. Finally, in vitro experiments demonstrated that knockdown of *TGIF1* significantly inhibited the proliferation and invasion of glioma cell. Taken together, our study, which is the first to comprehensively analyze *TGIF1* in gliomas, revealed it to be a novel oncogene in terms of its association with this disease. As such, *TGIF1* may be a potential therapeutic target for individualized treatment of patients with glioma.

## Graphical abstract

This study is the first to comprehensively analyze and reveal TGIF1, as a new oncogene, is closely related to the malignancy‐related phenotype of glioma, and that TGIF1 could be an independent risk factor for poor prognosis of glioma, which is expected to become a new target for individualized treatment of glioma patients.

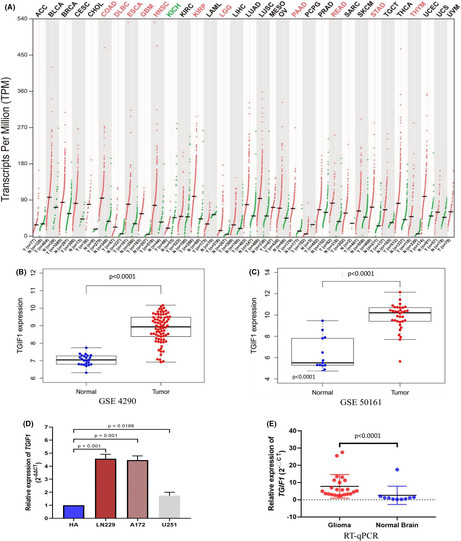

## 1 INTRODUCTION

Glioma is a malignant tumor of the central nervous system that exhibits high recurrence and fatality rates.^1^ Surgical resection followed by adjuvant chemoradiotherapy is widely prescribed by clinicians and has become the most effective treatment; moreover, novel therapies such as immunotherapy, photodynamic therapy, and transcranial magnetic stimulation adjuvant therapy continue to be developed.^2^, ^3^, ^4^ Nevertheless, the prognoses of patients with glioma have not substantially improved; this is largely owing to the aggressive growth of the tumor and its unclear boundaries, which results in low total resection and high recurrence rates. To improve treatment outcomes, the World Health Organization (WHO) incorporated molecular criteria into the revised classification system of glioma in 2016.^5^ This laid the foundation for enhancing the personalized treatment of patients with this disease, and emphasized the significance of molecular markers in their diagnosis, treatment, and prognosis.

Significant breakthroughs have been made in the basic and clinical research of glioma, and new diagnostic identifiers and treatment targets have been discovered and applied in clinical practice. Some patients who carry an *IDH1* mutation and 1p/19q co‐deletion have been found to have a better prognosis^6^, ^7^; moreover, anaplastic gliomas with *MGMT* promoter methylations are sensitive to radiotherapy and chemotherapy,^8^, ^9^ while mutations in the *PTEN* gene are useful for evaluating the prognoses of patients with anaplastic astrocytoma.^10^ Additionally, the mutation statuses of *BRAF* and *TP53*, as well as the expression of miR‐181d, have been useful for devising glioma treatment regimens and determining prognoses.^11^, ^12^ However, given that the outcomes of patients with this disease remain poor, screening for—and identifying—additional molecular markers of glioma is of far‐reaching significance for purposes of further understanding the biomolecular characteristics of this disease.

Transforming growth factor‐beta (TGF‐β) plays a key role in carcinogenesis^13^; it is produced and secreted by tumor cells and promotes their invasive and metastatic potentials.^14^ To date, a large number of studies found that TGF‐β‐induced factor homeobox 1 (*TGIF1*), a transcriptional repressor, plays an important biological role in various cancers. For example, silencing the *TGIF1* gene can inhibit the pathological progression of papillary thyroid cancer.^15^ Moreover, *TGIF1* can significantly promote the proliferation and invasion of gastric cells by participating in the XTP8/*TGIF1* signaling pathway.^16^ Another study suggested that the increased expression of *TGIF1* can promote the malignant progression of triple‐negative breast cancer cells and shorten the survival of patients with that disease.^17^ Although Shaw et al. found that the expression of *TGIF1* is downregulated in oligodendrogliomas with a 1p/19q co‐deletion,^18^ no comprehensive analyses of the relationship between *TGIF1* and the molecular and clinical characteristics of glioma, including the relevant cellular mechanisms, have been performed.

Hence, we performed this first study of its kind to systematically analyze *TGIF1* in glioma and elucidate its biological function as well as its role in patient prognosis using a large sample. Our findings suggest that *TGIF1* is a potentially novel molecular marker of glioma, and provide an additional understanding of the biomolecular characteristics of this disease, including its pathogenesis.

## 2 METHODS

### 2.1 Data collection and tissue sampling

Genomic data from thousands of gliomas were collected from the Chinese Glioma Genome Atlas (CGGA; http://www.cgga.org.cn/) database. We also downloaded RNA sequencing (RNA‐seq) and microarray data of 1018 and 301 gliomas, respectively, from the CGGA database. After excluding samples with incomplete clinical information, RNA‐seq, and microarray data from 748 to 268 gliomas, respectively, were used to investigate the role of *TGIF1* in this disease. We also acquired data pertaining to 698 glioma tissues from The Cancer Genome Atlas (TCGA; https://portal.gdc.cancer.gov/), which is a multi‐tumor cancer gene‐mapping program; we analyzed 653 of these samples for which complete clinical information was available to validate our findings using the CGGA.

Additionally, we analyzed the expression of *TGIF1* in 163 glioblastoma multiforme samples, 518 low‐grade gliomas, and 207 normal brain tissues from the Gene Expression Profiling Interactive Analysis (GEPIA2: http://gepia2.cancer‐pku.cn/), a large public online analysis tool that contains a large amount of gene expression data from tumor and corresponding normal tissues. We also used Oncomine (https://www.oncomine.org/resource/), a database for differential gene analysis that collects a large amount of tumor‐related microarray data, as a validation dataset for the differential expression of *TGIF1* in gliomas. Moreover, the microarray gene expression databases GSE4290 and GSE50161, which together contain 111 glioma and 36 normal brain tissues, were downloaded from the Gene Expression Omnibus (GEO: https://www.ncbi.nlm.nih.gov/geo/) database. All supported data we used can be reached on the GitHub page: https://github.com/Ligang111/2022‐03‐24.

### 2.2 Tissues and cell lines

Twenty‐three glioma and 10 paracancerous brain tissue samples were collected via surgical resection performed at Henan Provincial People's Hospital and immediately stored in liquid nitrogen. Histopathological diagnoses were independently assessed by two physicians, who provided consistent results. The samples used in this study were approved by the hospital ethics committee, and each participant signed an informed consent form before surgery. Human astrocytes (HA) and the glioma cell lines LN229, A172, and U251 were purchased from GenePharma (Suzhou, China). Cells were cultured in Dulbecco's Modified Eagle's Medium with 10% fetal bovine serum and 1% penicillin and were cultured in a sterile incubator containing 5% CO_2_ at 37°C.

### 2.3 Real‐time quantitative polymerase chain reaction

Total RNA from tissue samples and cells was extracted using Trizol (ThermoFisher Scientific), and the quality and quantity of total RNA were determined using Novoscript plus (catalog #E047; Novoprotein Scientific Inc.). Finally, we used 2× RealStar Green (catalog #A303‐05; GenStar) to conduct Real‐time quantitative polymerase chain reaction (RT‐qPCR). *GAPDH* was used as a housekeeping gene (forward: 5‐AAGAAGGTGGTGAAGCAGG‐3; reverse: 5‐GTCAAAGGTGG AGGAGTGG‐3). The primer sequences for *TGIF1* were forward: 5′‐GGATTGGCTGTATG AGCACCGT‐3; reverse: 5‐GCCATCCTTTCTCAGCATGT CAG‐3′.

### 2.4 Gene set enrichment analysis of *TGIF1*

After downloading CGGA RNA‐seq, CGGA microarray, and TCGA RNA‐seq data from their respective databases, the downloaded data were batch‐corrected and normalized using the ‘limma’ package of the R statistical software. Data from all three datasets were divided into two groups according to the median expression level of *TGIF1*. Gene set enrichment analysis (GSEA; version 4.0) was used to complete the functional enrichment analysis of *TGIF1*; after the data were uploaded, Kyoto Encyclopedia of Genes and Genomes cell signaling pathways were selected as the enrichment target path to analyze the three datasets. All enrichment analysis results were considered significantly enriched if *p*‐values were <0.05 and *q*‐values were <0.25.

### 2.5 Connectivity map analysis

Connectivity map (CMap; https://clue.io/) is a well‐known database in the field of pharmacogenomics. Based on CGGA RNA‐seq data, we used Pearson correlation analysis to identify genes that were associated with *TGIF1*. Two lists of genes, one positive and the other negative correlations with *TGIF1*, were uploaded to the CMap database; parameters with *p* < 0.01 and enrichment <−0.8 were used to identify associated small molecules.

### 2.6 Cell transfection

Based on the expression level of *TGIF1* in the cell lines, of which high expressed glioblastoma‐derived LN229 was used for in vitro experiments. The siRNA targeted on *TGIF1* was purchased from GenePharma. LN229 cells transfected with negative control siRNA (S: UUCUCCGAACGUGUCACGUTT, AS: ACGUGACACGUUCGGAGAATT) were set as the control group (siNC), and LN229 transfected with siRNA targeting *TGIF1* (S: GGAUGGCAAAGAUCCAAAUTT, AS: AUUUGGAUCUUUGCCAUCCTT) were set as the experimental group (siTGIF1). Then the mRNA level of *TGIF1* in both groups was tested by RT‐qPCR.

### 2.7 Cell proliferation assay

To test the effect of knocking down *TGIF1* on the proliferative capacity of glioma cells, the expression levels of the proliferation‐associated antigen Ki67 in different subgroups were first verified. After LN229 transfection of siRNA for 36 h, the cells were fixed and permeabilized and then blocked using 10% serum, followed by incubation using Ki67‐specific antibodies (Abcam, 1:200). After incubation with the specific secondary antibody, DAPI staining was performed. Furthermore, transfected LN229 cells were planted in 96‐well plates at a density of 2000 cells per well, and CCK8 solution was added at the set detection time points, and the absorbance value at 450 nm was detected after continued incubation for 4 h. Meanwhile, transfected LN229 cells were planted in a six‐well plate at a density of 500 cells per well. After continuing the culture for 14 days, the cell colonies were stained using crystal violet solution.

### 2.8 Cell invasion assay

Wound healing assay and Transwell assay were used to examine the effect of *TGIF1* on the invasive ability of glioma cells. After the full growth of transfected cells in the six‐well plate, a wound was made using a sterile pipette tip, and the culture was continued using serum‐free DMEM medium after PBS washing, and relative distances of the wound at the same location were photographed at the set time points. In addition, a certain amount of transfected cells were added to Transwell chambers and cultured using 5% serum medium. The lower chamber was added to the medium with 20% serum and continued to incubate for 48 h, and then the cells invading to the lower wall of Transwell chambers were stained using crystal violet solution.

### 2.9 Meta‐analysis of *TGIF1*

In order to collect more evidence to verify the impact of *TGIF1* on the prognosis of glioma patients, the study collected seven datasets including 1914 glioma patients (TCGA RNA‐seq: 653; CGGA microarray: 268; CGGA RNA‐seq: 748; GSE43378: 50; GSE4412: 85; GSE74187: 60; GSE83300: 50). These data were used for a meta‐analysis to verify the effect of *TGIF1* on the prognosis of glioma. First of all, the above seven datasets were subjected to a Cox analysis one by one to verify the impact of *TGIF1* on the prognosis of glioma patients. The *Q*‐test was used to verify the heterogeneity among the seven datasets, and the random‐effect model was used to complete the meta‐analysis based on *I* ^2^ = 92% and *p* < 0.01.

### 2.10 Statistical analysis

Statistical analyses were performed using the R software (version 3.6.3). The relationships between patient characteristics and *TGIF1* expression were evaluated using the Wilcoxon or Kruskal–Wallis tests. Kaplan–Meier and Cox regression analyses were used to analyze the effect of *TGIF1* overexpression on the survival of patients with glioma. The experimental data were analyzed by one‐way ANOVA, and all statistical analyses were processed by SPSS 22.0. Two‐sided *p*‐values <0.05 were considered statistically significant.

## 3 RESULTS

### 3.1 *TGIF1* was significantly increased in gliomas

**FIGURE 1:**
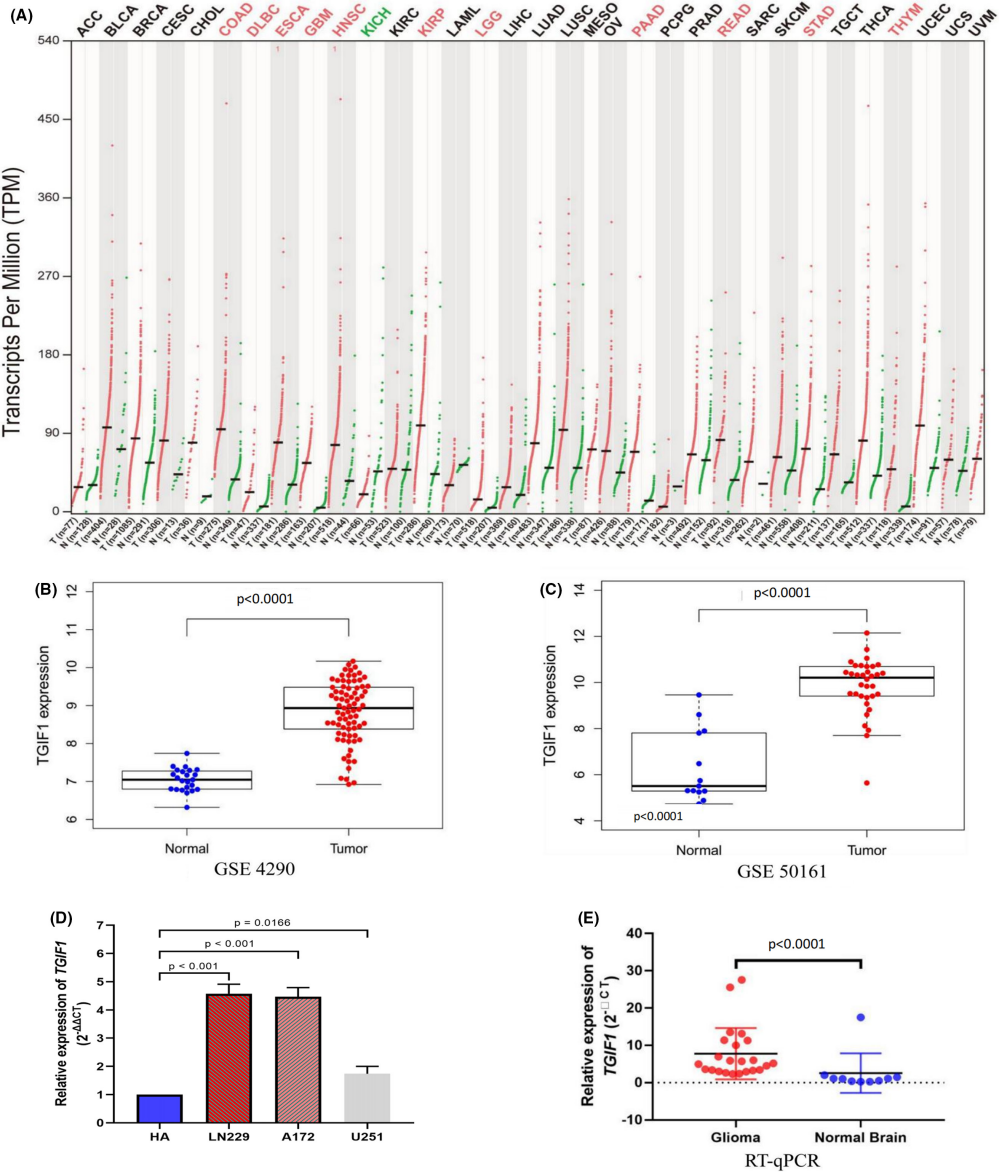
*TGIF1* is highly expressed in gliomas. (A) Analysis of the GEPIA database revealed that *TGIF1* is expressed in various tumors. Red represents an increased expression of *TGIF1* relative to the corresponding control, while green represents decreased *TGIF1* expression. (B) *TGIF1* expression in normal brain tissues and glioma tissues according to the GSE4290 dataset. (C) *TGIF1* expression in normal brain tissues and glioma tissues according to the GSE50161 dataset. (D) The expression levels of *TGIF1* mRNA in human astrocytes and glioblastoma cell line LN229, A172, and U251. (E) *TGIF1* expression in normal brain and glioma tissues collected from clinical patients. *p* value less than 0.05 is statistically significant

We first analyzed the expression of *TGIF1* in tumors using the GEPIA and Oncomine databases and found that this gene's expression was higher in central nervous system tumors than in normal brain tissues in both databases (Figure 1A and Figure S1). Further exploration of different GEO and Oncomine datasets revealed that *TGIF1* was significantly overexpressed in gliomas compared to normal tissue (Figure 1B,C and Figure S1). RT‐qPCR confirmed that the mRNA expression levels of *TGIF1* in glioma cell lines and tissues were markedly increased over controls (Figure 1D,E). These results laid the foundation for further studying the role of *TGIF1* in glioma.

### 3.2 *TGIF1* is correlated with the clinical characteristics of glioma

**FIGURE 2:**
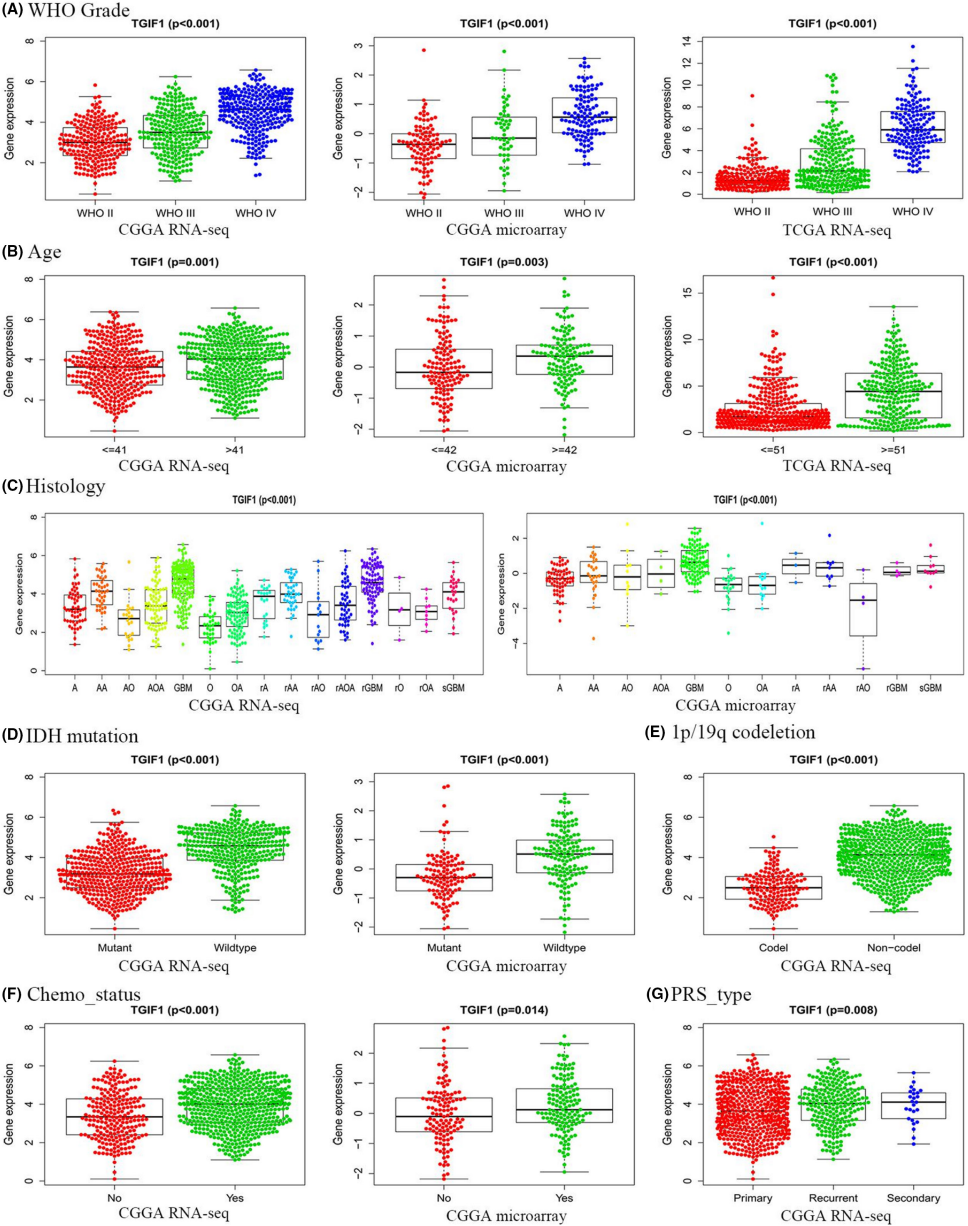
Correlation between *TGIF1* expression and molecular/clinical features associated with glioma. (A) *TGIF1* expression in gliomas of various World Health Organization grades. (B) The relationship between *TGIF1* expression and age in patients with glioma. (C) *TGIF1* expression in gliomas of different histological subtypes. (D) The relationship between *TGIF1* expression and *IDH* mutation status. (E) The relationship between *TGIF1* expression and 1p/19q co‐expression status. (F) Differential analysis of *TGIF1* expression in gliomas treated with chemotherapy after surgery. (G) *TGIF1* expression in primary, recurrent, and secondary gliomas. *p* value less than 0.05 is statistically significant. Chemo, chemotherapy; IDH, isocitrate dehydrogenase; PRS, primary‐recurrent‐secondary; WHO, World Health Organization

TCGA‐RNA‐seq, CGGA‐RNA‐seq, and CGGA‐microarray data were grouped according to the different grades of gliomas. As shown in Figure 2A, the expression level of *TGIF1* was highest in WHO grade IV gliomas and was lower in WHO grade III tumors and lowest in WHO grade II gliomas. Additionally, recurrent gliomas showed higher expression levels of *TGIF1* than primary gliomas (Figure 2G); this phenomenon was observed in several histological subtypes (Figure 2C). These findings suggest that *TGIF1* may be involved in the malignant progression of glioma. We also found that the expression levels of *TGIF1* in elderly patients, patients with *IDH* wildtype, and patients with 1p/19q non‐codeletion were higher than in the corresponding control groups (Figure 2B,D,E). *IDH* mutation and 1p/19q co‐deletion are known to be favorable prognostic indicators in patients with glioma. In contrast, our data indicated that *TGIF1* promotes the malignant progression of gliomas and is associated with poor prognosis.

### 3.3 Effect of high expression of *TGIF1* on overall survival and diagnostic value of prognosis of glioma patients

**FIGURE 3:**
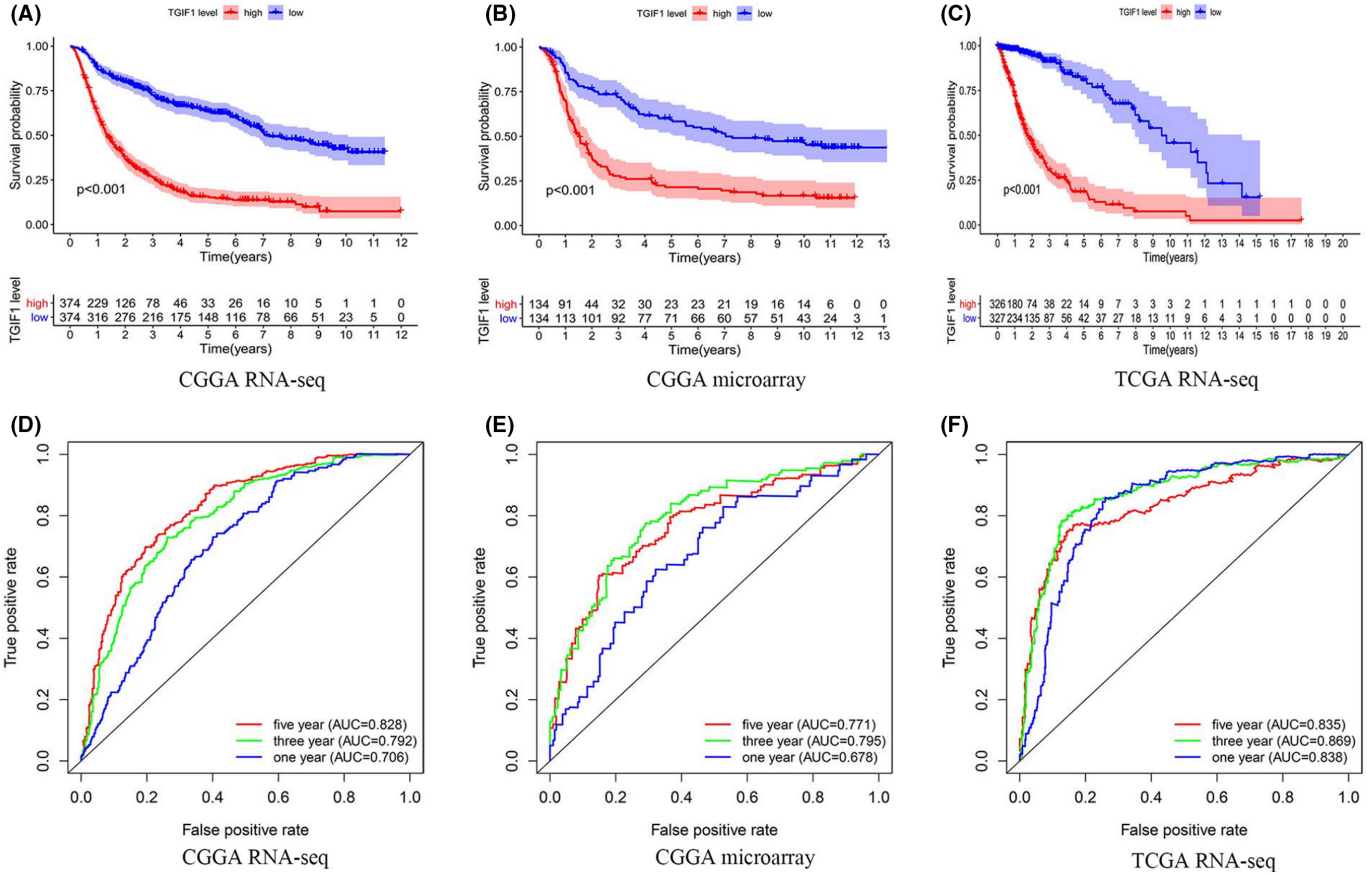
The relationship between *TGIF1* expression and overall survival of patients with glioma. (A–C) Correlations between *TGIF1* expression levels and overall survival of glioma patients based on the CGGA RNA‐seq dataset, CGGA microarray dataset, and TCGA RNA‐seq dataset, respectively. (D–F) Receiver operating characteristic curves based on the CGGA RNA‐seq, CGGA microarray, and TCGA RNA‐seq datasets. *p <* 0.05 is statistically significant, AUC > 0.7 is considered credible

To understand the role of *TGIF1* expression in the survival of patients with glioma, we divided the samples derived from the aforementioned databases into high‐ and low‐expression groups based on the median expression level of *TGIF1* in the samples and used the Kaplan–Meier method to explore the relationship between this gene's expression and patient survival. When considering all grades of gliomas collectively, higher expression levels of *TGIF1* correlated with shorter overall survival (Figure 3A–C). We then divided the patients into high‐grade (III and IV) and low‐grade (II) gliomas to evaluate the effect of *TGIF1* on their prognoses; a favorable effect of low *TGIF1* expression on the prognosis of patients with low‐grade glioma was only observed when analyzing the RNA‐seq data from the CGGA and TCGA (Figure S2A–C). However, three different datasets consistently showed that high expression of *TGIF1* was associated with a reduced overall survival time among patients with high‐grade glioma (Figure S2D–F).

Given the influence of *IDH* mutations on prognosis, we also investigated the impact of *TGIF1* on the prognosis of patients with varying *IDH* mutation statuses. According to the CGGA data, the high expression of *TGIF1* significantly shortened the overall survival of patients with *IDH* mutations regardless of glioma grade and 1p19q codeletion (Figure S2H–I and K). Finally, the high expression of *TGIF1* did affect the prognoses of patients of any glioma grade who had wildtype *IDH* regardless of 1p19q codeletion (Figure S2G and J).

The area under the receiver operating characteristic curve suggested that *TGIF1* expression carries a high sensitivity for predicting the prognoses of these patients (Figure 3D–F). Additionally, according to the grouping of all subtypes in survival analysis, *TGIF1* in all subtypes has diagnostic value for the prognosis of patients (Figure S3). The area under the curve (AUC) was greater than 0.7 in multiple points in time. Therefore, it can be concluded that the increased expression of *TGIF1* has a significant impact on the overall survival of patients with gliomas and has diagnostic value, especially in high‐grade glioma.

### 3.4 *TGIF1* is an independent predictor of poor prognosis in patients with glioma

**FIGURE 4:**
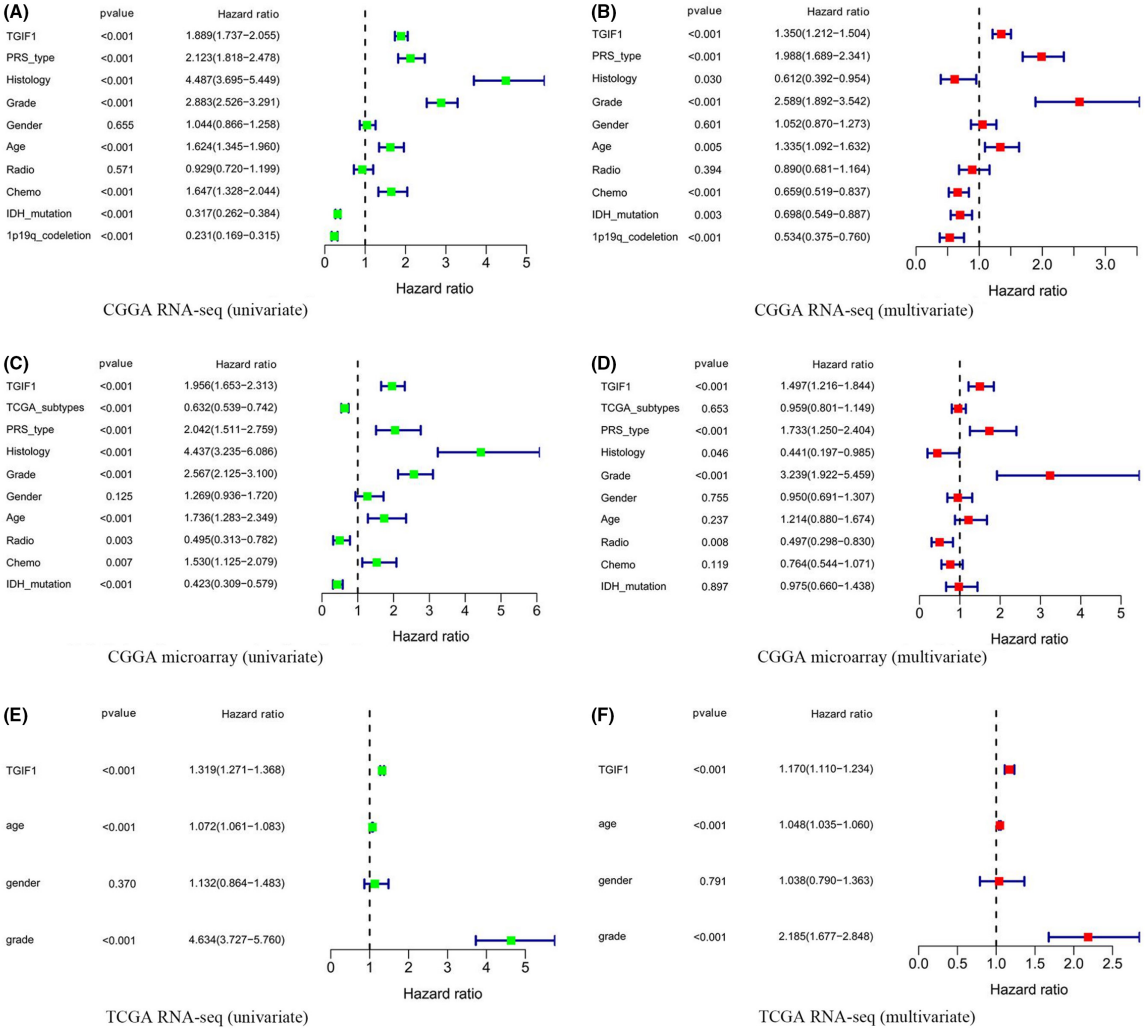
Prognostic value of various factors of patients with glioma. (A, B) Univariate and multivariate analyses based on the CGGA RNA‐seq dataset. (C, D) Univariate and multivariate analyses based on the CGGA microarray dataset. (E, F) Univariate and multivariate analyses based on TCGA RNA‐seq dataset. The range of hazard ratio less than 1 is considered as a protective factor; The range of HR more than 1 is considered as a risk factor. *p* < 0.05 considered statistically significant. PRS, primary‐recurrent‐secondary; radio, Radiotherapy; chemo, Chemotherapy; IDH, isocitrate dehydrogenase

To determine whether *TGIF1* expression is an independent predictor of patient survival, we established Cox proportional hazards models based on the different datasets. Univariate and multivariate analyses suggested that *TGIF1* expression is an independent negative prognostic risk factor for patients with all grades of gliomas (hazard ratio [HR] >1) (Figure 4). We then divided the patients into high‐grade (III and IV) and low‐grade (II) gliomas to evaluate the risk of *TGIF1* on the prognosis of patients; the expression of *TGIF1* has a significant risk for the prognosis of patients with low‐grade glioma was only observed when analyzing the RNA‐seq data from the CGGA and TCGA (HR >1) (Figures S4A–C and S5A–C). However, three different datasets consistently showed that the expression of *TGIF1* has a significant risk for the prognosis of patients with high‐grade glioma (HR>1) (Figures S4D–F and S5D–F). More importantly, no matter which group *TGIF1* is in, it shows that the high expression of *TGIF1* is a risk to the prognosis of glioma patients according to the molecular subtypes of gliomas (HR >1) (Figures S4G–K and S5G–K). Mutual validation among the above databases confirmed that *TGIF1* was an independent risk factor for the prognosis of glioma patients, especially high‐grade glioma.

### 3.5 Biological function of *TGIF1* in glioma

**FIGURE 5:**
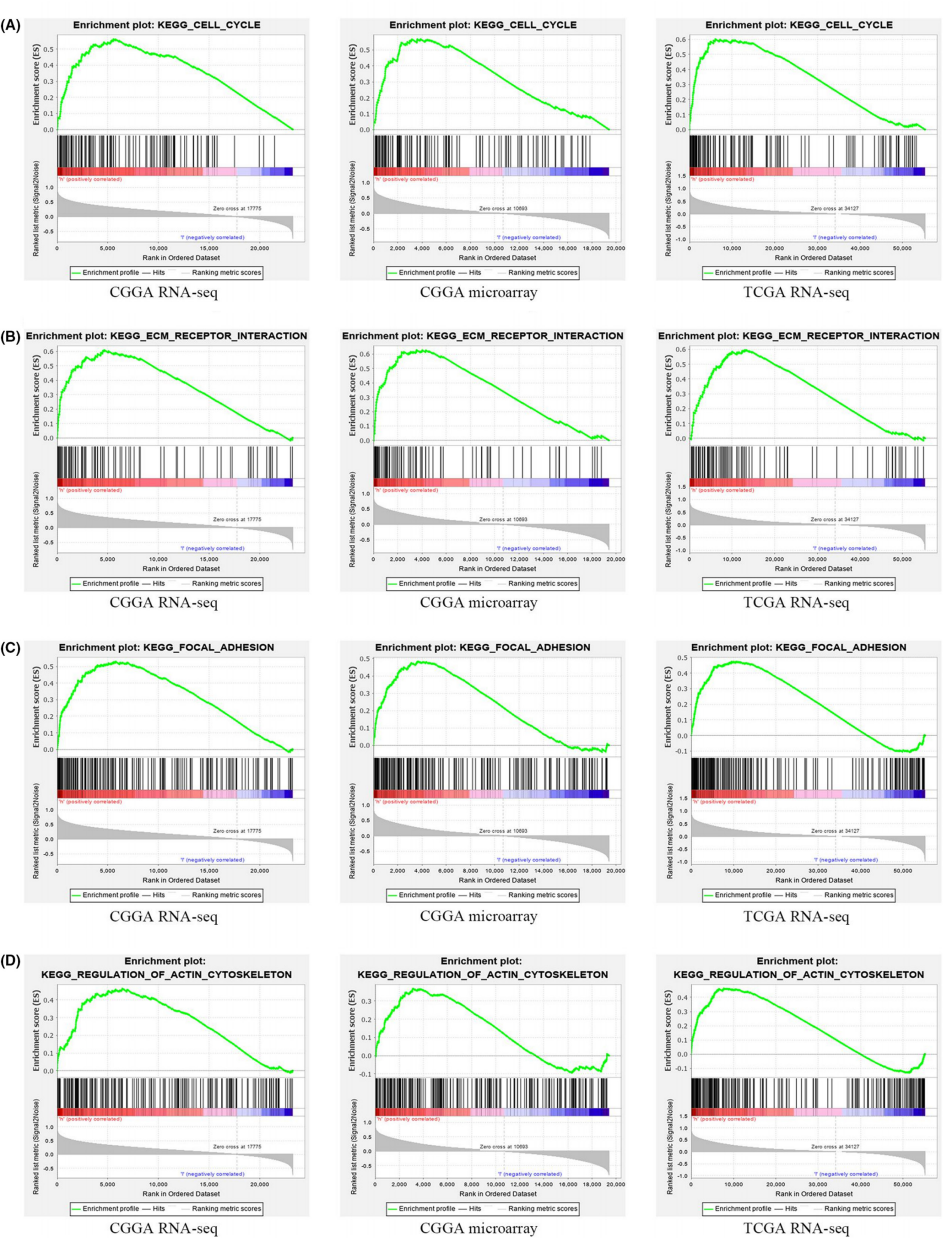
The enrichment of *TGIF1* gene in signaling pathways based on gene set enrichment analyses of the CGGA RNA‐seq, CGGA microarray, and TCGA RNA‐seq datasets. (A) Enrichment in cell cycle signaling pathway; (B) Enrichment in extracellular matrix receptor interaction signaling pathway. (C) Enrichment in focal adhesion signaling pathway. (D) Enrichment in actin cytoskeleton signaling regulatory pathway. *p* < 0.05 and FDR *q*‐value <0.25 were considered as significantly enriched. See Table 1 for more details

Given our evidence that *TGIF1* may be involved in the pathological processes of glioma, we further explored the cellular mechanisms potentially involved using GSEA. Several signaling pathways involved in cancer progression were identified, including those related to actin cytoskeleton rearrangement, extracellular matrix (ECM)‐receptor interaction, cell cycle regulation, and focal adhesion formation (Figure 5, Table 1).

**TABLE 1:** The gene set enriches the high *TGIF1* in three databases

Gene set name	CGGA RNA‐seq			CGGA microarray			TCGA RNA‐seq		
	NES	NOM *p*‐val	FDR *q*‐val	NES	NOM *p*‐val	FDR *q*‐val	NES	NOM *p*‐val	FDR *q*‐val
Regulation of actin cytoskeleton	1.72	0.00	0.14	1.52	0.04	0.14	1.77	0.01	0.04
ECM receptor interaction	1.86	0.00	0.11	1.78	0.01	0.08	1.86	0.00	0.04
Cell cycle	1.68	0.04	0.10	1.73	0.04	0.08	1.86	0.01	0.04
Focal adhesion	1.88	0.00	0.13	1.76	0.02	0.09	1.67	0.01	0.06

*Note*: Gene sets with NOM *p* < 0.05 and FDR *q*‐value <0.25 were considered as significantly enriched.

Abbreviations: FDR: false discovery rate; NES, normalized enrichment score; NOM, nominal.

### 3.6 Analysis of genes co‐expressed with *TGIF1*

**FIGURE 6:**
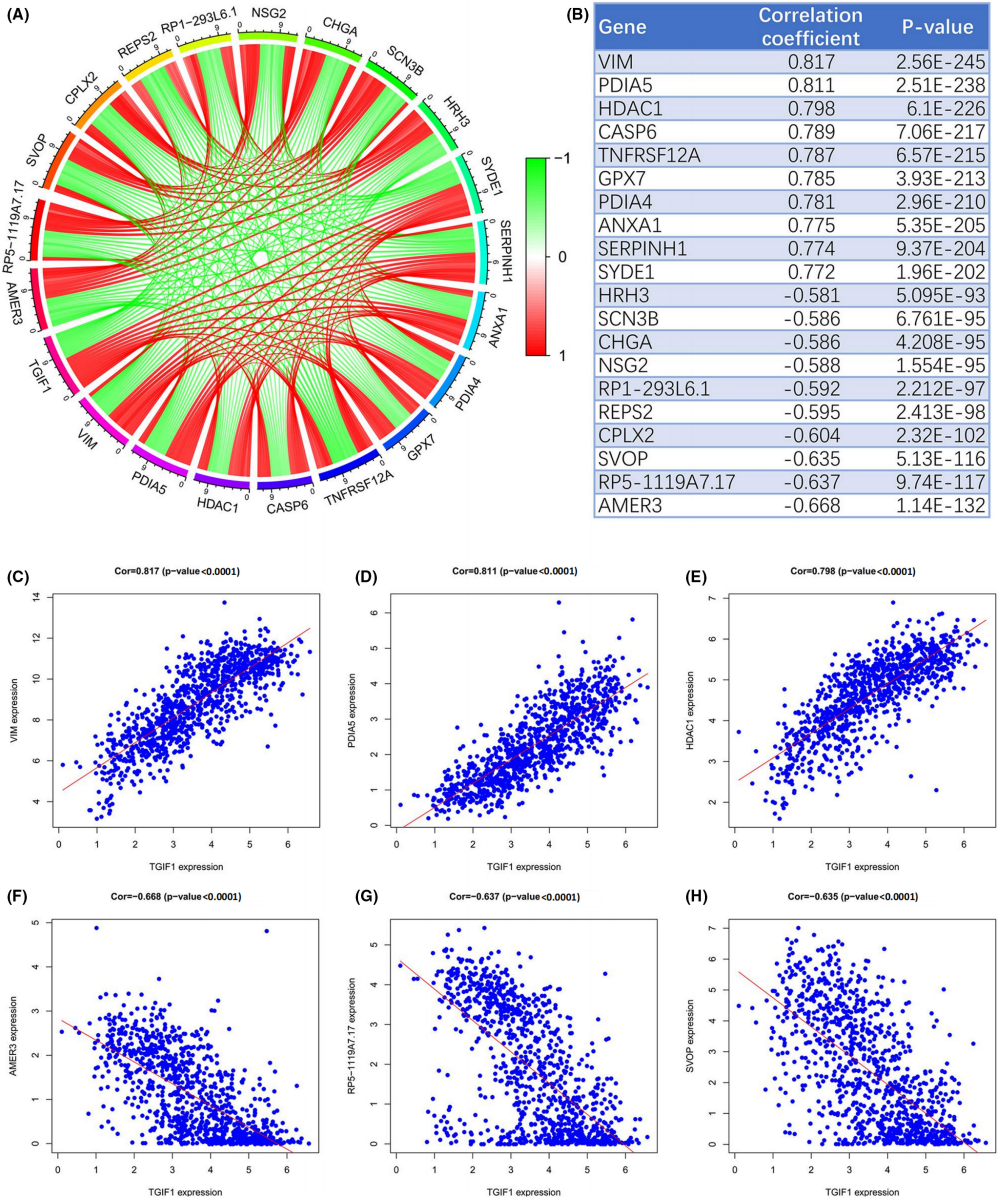
Analyses of *TGIF1* co‐expression with other genes. (A) Circle plot shows 10 upregulated and 10 downregulated genes whose expression levels were significantly associated with *TGIF1*. (B) Enrichment parameters of the 20 genes whose expression levels are significantly associated with *TGIF1*. (C–E) Correlation analysis between *VIM*, *PDIA5*, *HDAC1*, and *TGIF1*. (F–H) Correlation analysis between *AMER3*, *RP5‐1119A7.17*, *SVOP*, and *TGIF1*. *p <* 0.05 is statistically significant

Owing to the complexity of the mechanisms governing the malignant progression of glioma, other factors may be involved in its development. Pearson's correlation analysis was used to analyze the CGGA RNA‐seq data to identify other genes associated with *TGIF1* in glioma tissues. Circle plots revealed the interconnections among the 20 genes most related to *TGIF1* (Figure 6A,B); *VIM*, *PDIA5*, *HDAC1*, *CASP6*, *TNFRSF12A*, *GPX7*, *PDIA4*, *ANXA1*, *SERPINH1*, and *SYDE1* were positively correlated with *TGIF1* (Figure 6C–E and Figure S6A–G), while *HRH3*, *SCN3B*, *CHGA*, *NSG2*, *RP1‐293 L6.1*, *REPS2*, *CPLX2*, *SVOP*, *RP5‐1119A7.17*, and *AMER3* were negatively correlated (Figure 6B,F–H and Figure S6H–N). These results further suggest that *TGIF1* does not exert its tumorigenic effects in gliomas via a single signaling pathway; rather, they highlight the multifaceted pathways of this process.

### 3.7 Identification of small molecules targeting *TGIF1*

**FIGURE 7:**
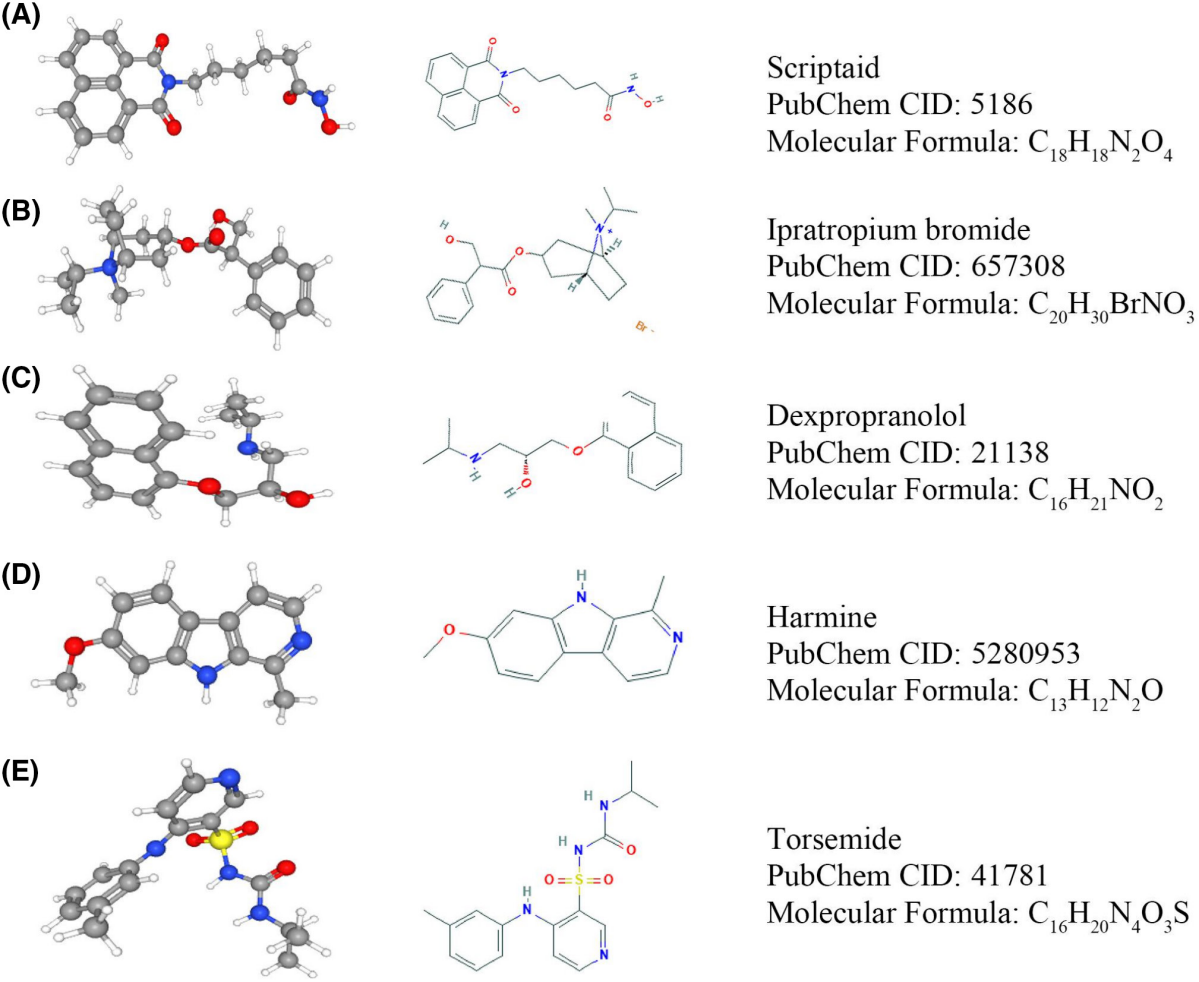
Two‐ and three‐dimensional structures of anti‐glioma small molecule drugs targeting *TGIF1*. (A) Scriptaid, (B) Ipratropium bromide, (C) Dexpropranolol, (D) Harmine, and (E) Torasemide

We identified five small molecule compounds with potential value in treating glioma using the CMap online analysis tool; their corresponding parameters are shown in Table 2. Moreover, their two‐ and three‐dimensional structures were determined using the PubChem online tool (https://pubchem.ncbi.nlm.nih.gov) (Figure 7).

**TABLE 2:** Small molecule drugs targeting *TGIF1* against glioma

CMap name	Enrichment	*p* value
Scriptaid	−0.920	0.0009
Torasemide	−0.903	0.0002
Dexpropranolol	−0.865	0.0050
Ipratropium bromide	−0.846	0.0073
Harmine	−0.828	0.0017

### 3.8 To verify the role of *TGIF1* as a pathogenic molecule in the prognosis of glioma

**FIGURE 8:**
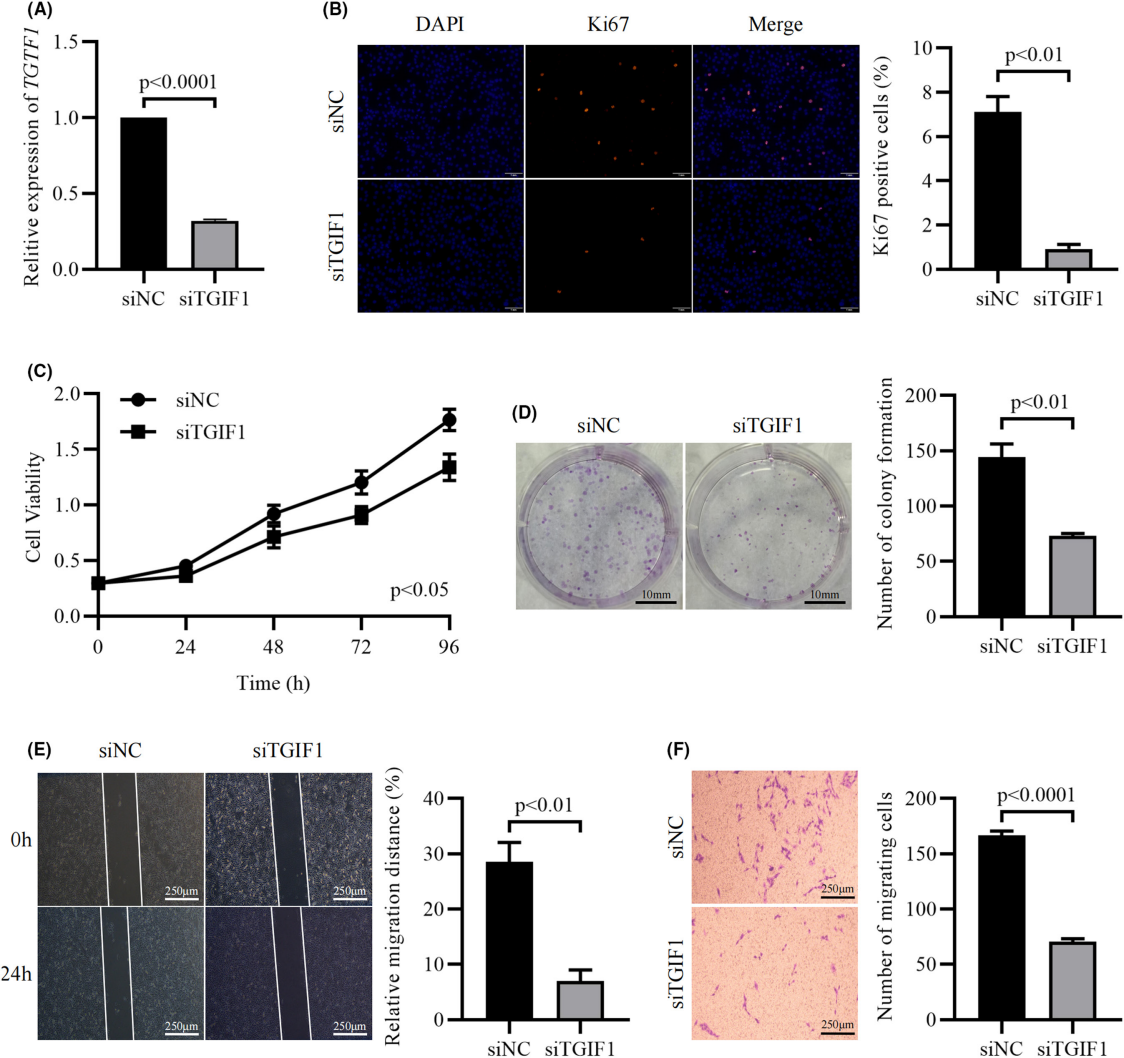
Knockdown of *TGIF1* significantly inhibits the proliferation and invasion of LN229 cells. (A) The expression of *TGIF1* in both siNC and siTGIF1 group. (B) Staining of Ki67 positive cells in both groups. (C) Cell viability of LN229 cells in siNC and siTGIF1 group at different time points. (D) Staining of cell colonies in both groups. (E) Relative distances of the wound healing assay. (F) Staining of invasive LN229 cells in the Transwell assay. *p <* 0.05 is statistically significant

In the cells of the experimental group, the expression level of *TGIF1* was significantly less than that of the control group (*p* < 0.0001) (Figure 8A). The proportion of Ki67‐stained positive cells was significantly lower in the siTGIF1 group than that in the siNC group (*p* < 0.01) (Figure 8B). Besides, CCK8 assay showed that the cell viability of LN229 cells in the experimental group was lower than that of the control group at 24 h (*p* < 0.05), 48 h (*p* < 0.05), 72 h (*p* < 0.01), and 96 h (*p* < 0.01) (Figure 8C). Meanwhile, the number of cell colonies formed in the experimental group was significantly less than that in the control group (*p* < 0.01) (Figure 8D). Furthermore, compared to the control group, the invasive ability of LN229 cells in the experimental group was significantly reduced as confirmed by wound healing assay (*p* < 0.01) (Figure 8E) and Transwell assay (*p* < 0.0001) (Figure 8F). Therefore, it can be confirmed that the high expression of *TGIF1* can promote the malignant behavior of glioma cells. in order to try to find more evidence to confirm that *TGIF1* is a pathogenic molecule for the prognosis of patients with glioma, the study used a meta‐analysis to find that each data showed that *TGIF1* was a risk for the prognosis of patients in seven independent datasets and the pooled HR for the association between *TGIF1* expression and OS in patients was 1.61 (1.32–1.96). Thus, *TGIF1* is a reliable pathogenic factor for glioma (Figure S7).

## 4 DISCUSSION

A large number of studies have found that *TGIF1* plays a critical biological function in a variety of cancers and that its expression is associated with the enhanced invasion, migration, and metastasis of tumor cells. However, systematic studies of *TGIF1* in gliomas have not been performed to date. In our present analysis of glioma samples using public databases, we found an association between *TGIF1* expression levels and unfavorable clinical features. We also clarified biological mechanisms of *TGIF1* involvement in glioma using GSEA.

Using the online GEPIA tool, we discovered that the expression levels of *TGIF1* in numerous tumor tissues such as glioblastoma multiforme and low‐grade glioma were higher than those in the matched normal tissues; this was also verified using two GEO datasets (GSE4290 and GSE50161). Previous studies have shown that elevated *TGIF1* in triple‐negative breast cancer can promote malignant progression of the tumor and shorten the patients' survival.^17^ Our analysis of the CGGA RNA‐seq data showed that increased expression of *TGIF1* can shorten the survival of patients with glioma regardless of disease grade; this was verified when examining the CGGA microarray and TCGA RNA‐seq data. WHO grade IV gliomas are the most malignant and are associated with shorter median and overall survival times, and patients with gliomas carrying *IDH* mutations and 1p/19q co‐deletions achieve better survival than do those with *IDH* wildtype and no 1p/19q co‐deletions.^6^, ^7^ To investigate the impact of *TGIF1* expression on the survival of patients with glioma, we analyzed the correlation between *TGIF1* mRNA levels and clinical features associated with glioma prognosis. *TGIF1* mRNA was markedly elevated in highly malignant gliomas compared to low‐grade counterparts; moreover, the expression of *TGIF1* in recurrent gliomas was higher than that in primary gliomas. We also found that the expression level of *TGIF1* in *IDH*‐mutated and 1p/19q co‐deleted tissues were lower than that in the matched control group. These results suggest that *TGIF1*, which is increasingly expressed commensurate with higher stage disease, may play an active role in glioma tumorigenesis and progression while being a negative prognostic factor. Our Cox analysis further revealed that *TGIF1* is an independent predictor of the prognosis of patients with glioma.

GSEA revealed that actin cytoskeleton regulation, ECM receptor interaction, cell cycle regulation, and focal adhesion signaling pathways (all of which play important roles in tumor proliferation, migration, and invasion) were remarkably enriched in patient samples with higher *TGIF1* mRNA; this was validated in different datasets. Previous studies have found that actin cytoskeleton regulation is critical for the proliferation and invasion of glioma cells.^19^ Jiang et al. also found that cellular ECM receptors regulate glioma invasion and metastasis using transcriptome integration analysis,^20^ while other investigators found that cell cycle protein alterations are involved in glioma cell proliferation and apoptosis^21^, ^22^ and that the dysregulation of focal adhesion assembly can promote cancer cell metastasis resistance to chemoradiotherapy.^23^, ^24^ As such, *TGIF1* (as a transcriptional regulator) appears to have a multifaceted role in the proliferation, differentiation, metastasis, invasion, and apoptosis of glioma cells, thereby illustrating the complexity of glioma tumorigenesis.

We also found that there are many other genes co‐expressed with *TGIF1* that may contribute to the malignant progression of glioma. For example, protein disulfide isomerase family A member 4, which is encoded by a gene (*PDIA4*) whose expression is directly correlated with *TGIF1*, can disrupt the DNA repair mechanism, thereby promoting the proliferation and metastasis of cancer cells.^25^, ^26^ ‘RALBP1‐associated Eps domain containing 2’, whose encoding gene *REPS2*’s expression levels are inversely correlated with those of *TGIF1*, blocks the proliferation and migration of tumor cells and inhibits their apoptosis, and is therefore an indicator of favorable prognosis as related to prostate, breast, and esophageal cancers.^27^, ^28^, ^29^ These findings strongly indicate that *TGIF1* is an oncogene that promotes tumor progression and shortens patient survival by participating in different tumorigenic biological processes. Moreover, our results provide a rationale for developing therapeutic agents that target *TGIF1*.

The CMap online analysis tool identified five small molecules that are potentially potent against *TGIF1*. The anticancer properties of these drugs have already been demonstrated in previous studies. For example, scriptaid, which is an inhibitor of histone deacetylase, can induce apoptosis of glioma cells by activating Jun N‐terminal kinase.^30^, ^31^ Dexpropranolol is a well‐characterized blocker of non‐selective beta‐adrenergic receptors, and several studies have shown that it exhibits anti‐tumor properties in gastric, prostate, and breast cancers.^32^, ^33^, ^34^ Harmine, a natural plant extract that has been shown to have anticancer characteristics in numerous tumor types, depletes stem‐like cells in glioblastoma by inhibiting Akt phosphorylation.^35^, ^36^, ^37^ The repurposing of traditional medicines continues to gain attention, and some previously released drugs have demonstrated good clinical efficacy in the treatment of new diseases. For example, aspirin, which is widely prescribed to treat antipyretic analgesia, has been used to prevent transient cerebral ischemia attacks and myocardial infarction because of its inhibitory effect on platelet aggregation.^38^ Moreover, the lipid‐lowering agent atorvastatin has recently been used for the conservative treatment of chronic subdural hemorrhage.^39^ The global outbreak of the novel coronavirus (2019‐nCoV) in 2019 brought about a catastrophic pandemic owing to the lack of drugs or vaccines; however, the fact that old drugs such as ribavirin, chloroquine, and hydroxychloroquine have played important roles in saving patients' lives illustrates the importance of novel uses of traditional medicines for combating diseases.^40^, ^41^, ^42^

Furthermore, this study confirmed the effect of *TGIF1* on glioblastoma cells through in vitro experiments. As a hallmark feature of malignant tumors, unlimited malignant proliferation is a fundamental factor in the rapid development of glioma and tumor recurrence.^43^ Many of the researches on glioma have focused on the proliferative capacity of tumor cells, such as overexpression of miR‐129‐5p can inhibit proliferation of LN229 cells and blocked the cell cycle in G1 Phase.^44^ In the present study, it is similarly demonstrated that *TGIF1* can significantly affect the proliferative capacity of gliomas, suggesting that *TGIF1* serves as a clearly therapeutic target to inhibit glioma proliferation. In addition, the fact that tumors invade into normal tissues and cause indistinct tumor margins, thus inducing tumor recurrence recognizes invasion as another feature of malignant glioma.^45^ It is reported that miR‐128 can significantly decrease the stability of COX‐2 mRNA and inhibit proliferation and invasion of LN229 cells.^46^ Also, neural‐specific ECM molecule BEHAB/Brevican affects the invasion of glioma cells through the mediation of the extracellular matrix.^47^ The present study also confirmed that *TGIF1* can significantly affect the invasive ability of glioma by wound healing assay and transwell assay. At this point, the critical role of *TGIF1* in the malignant progression of glioma has been clearly demonstrated. Combined with the above bioinformatics results, a conclusion is reached that *TGIF1* can serve as a diagnostic and therapeutic target for glioma.

There were some limitations to this study. First, because various datasets have different categories of clinical information of patients, the clinical features of some patients with glioma could not be fully verified. These included a lack of data on the original recurrence status of tumors in the CGGA microarray and TCGA datasets, as well as incomplete data on glioma tissue subtypes in TCGA datasets. Additionally, the lack of information on gene expression profiles after chemoradiotherapy precluded further studies of the effect of chemoradiotherapy on *TGIF1* expression. However, the correlation observed between chemotherapy and *TGIF1* expression in this study further illustrated that tissues of greater malignant potentials have higher levels of *TGIF1*. Patients who receive additional chemotherapy owing to residual postoperative pathological findings generally have tumors with a higher degree of malignancy.

## 5 CONCLUSION

We found that the high expression of *TGIF1* in gliomas is closely correlated with clinical characteristics associated with poor prognosis. Moreover, *TGIF1* was found to be an independent predictor of patient prognosis. Finally, knockdown of *TGIF1* significantly inhibits the proliferation and invasion of glioma cell. Therefore, *TGIF1* appears to be a novel prognostic indicator and therapeutic target in patients with gliomas.

## CONFLICT OF INTEREST

All authors have no conflict of interest in the ownership of the study.

## AUTHOR CONTRIBUTIONS

Gang Li, Zhendong Liu, and Baoya Wang conceived and designed the experiment. Qiong Ma and Xuelin Wang analyzed and checked the data, Zhendong Liu, Baoya Wang, and Kunshan Guo wrote the manuscript. The final manuscript has been approved by the authors.

## ETHICAL APPROVAL

The study protocol was approved by the Ethics Committee of Henan Provincial People‘s Hospital (Zhengzhou, China).

## Supporting information


Figure S1

Figure S2

Figure S3

Figure S4

Figure S5

Figure S6

Figure S7

## Data Availability

The datasets used and analysed during the current study are available from the corresponding author on reasonable request.
