# Supplementary figures and images for: TGIF1 overexpression promotes glioma progression and worsens patient prognosis
Source: Cancer Med. 2022 May 15;11(24):5113–28. doi: 10.1002/cam4.4822 (PMC9761070; doi:10.1002/cam4.4822)

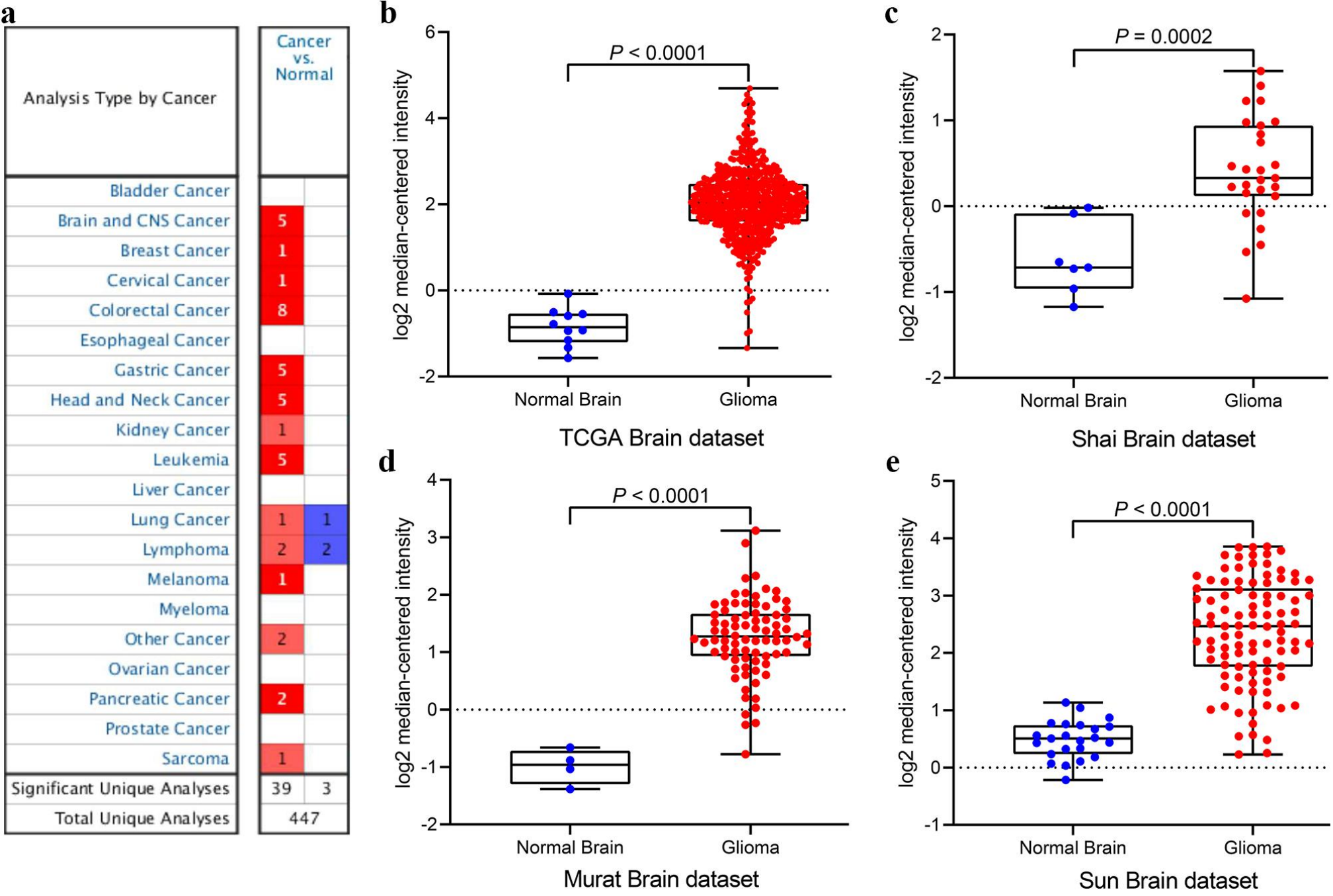

Supplement: Supplementary file 1 — Figure S1 [file CAM4-11-5113-s007.tif]

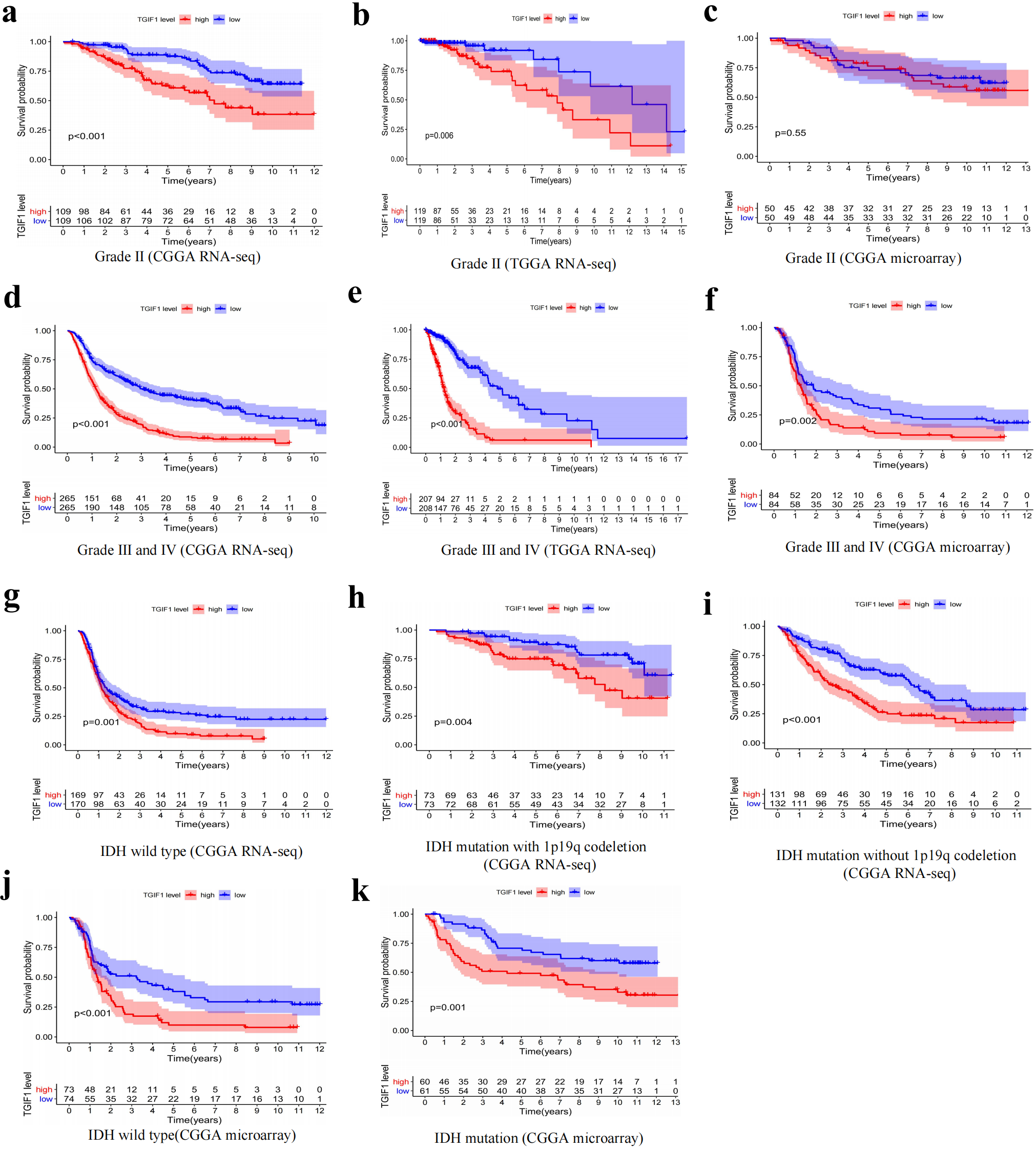

Supplement: Supplementary file 2 — Figure S2 [file CAM4-11-5113-s006.tif]

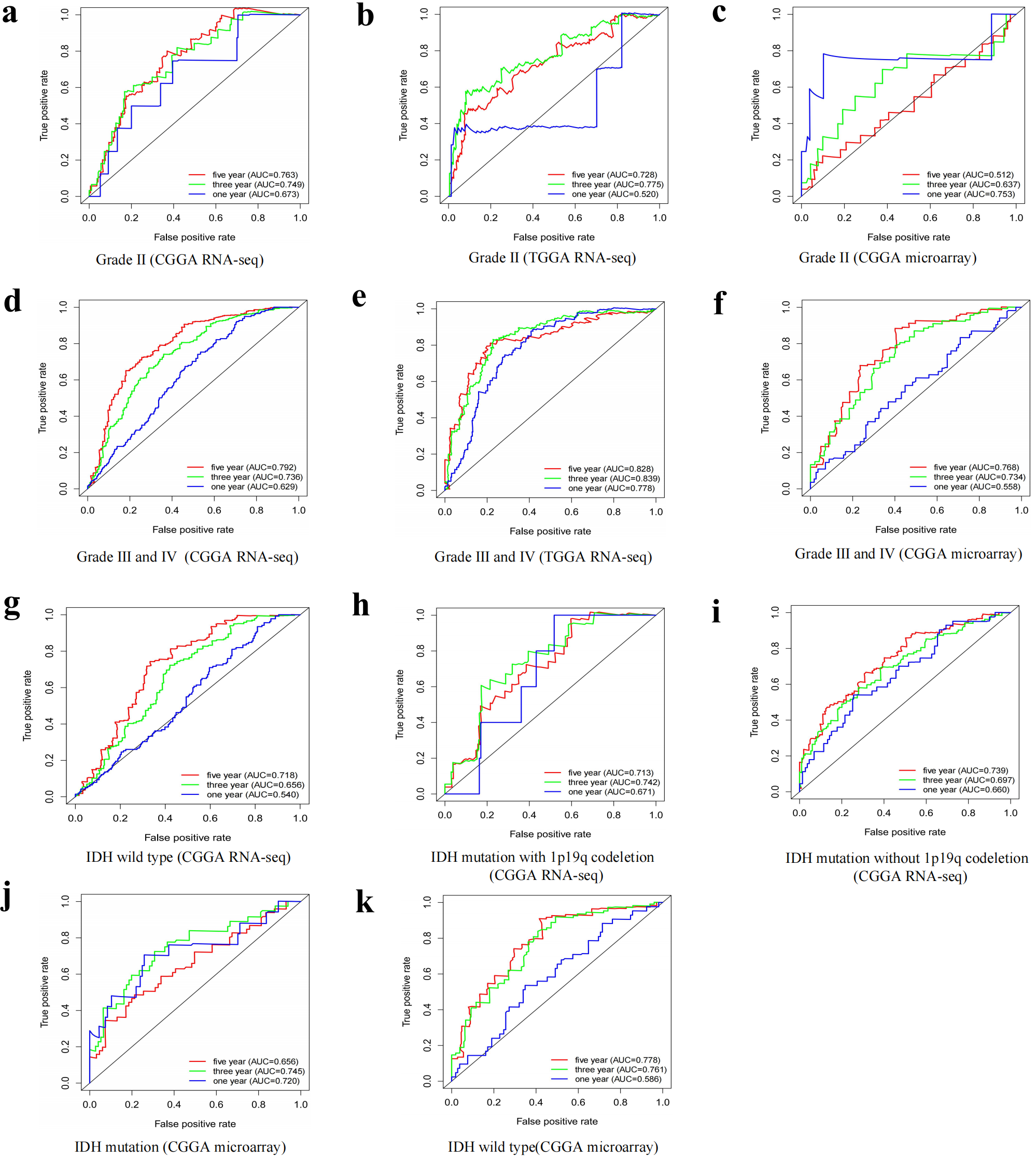

Supplement: Supplementary file 3 — Figure S3 [file CAM4-11-5113-s002.tif]

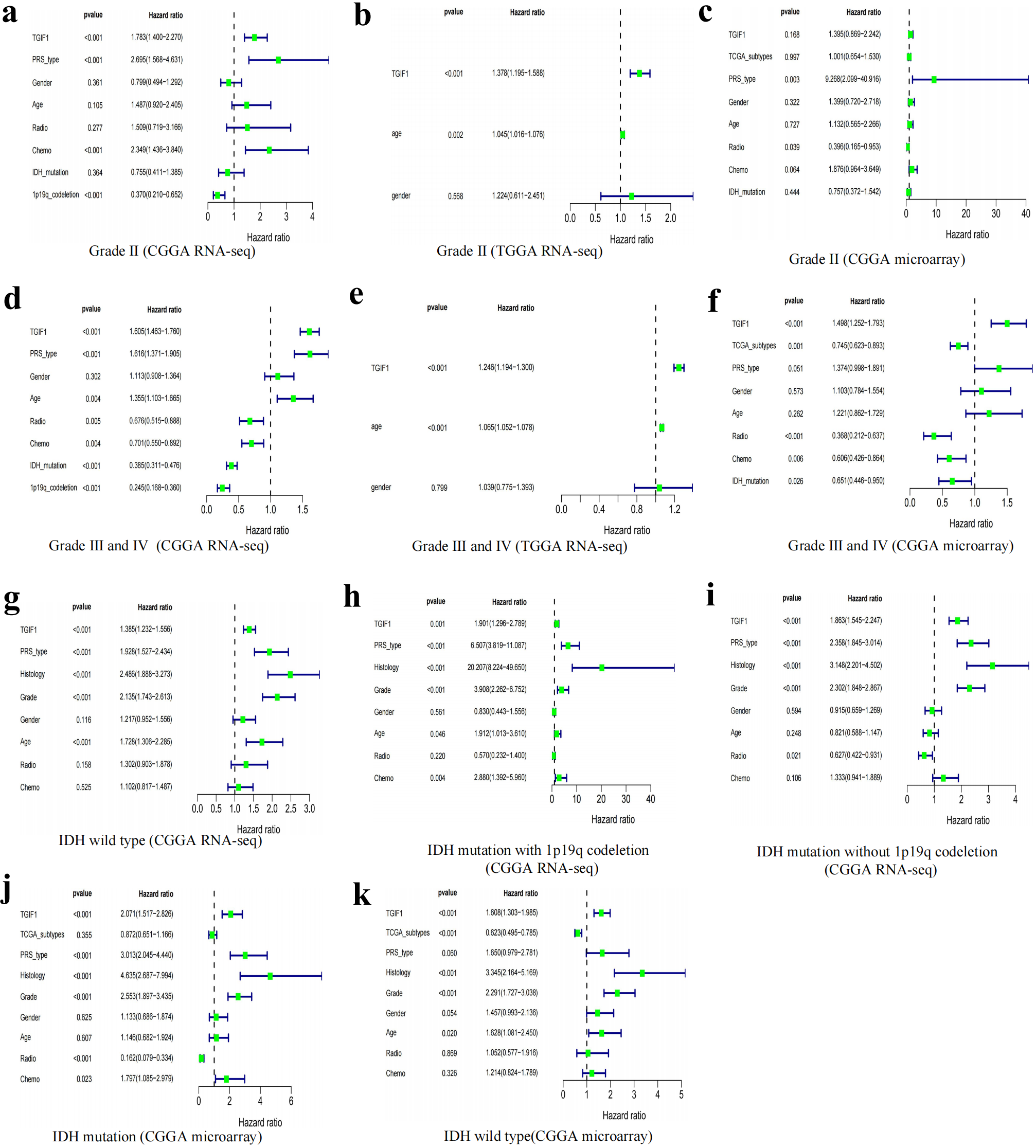

Supplement: Supplementary file 4 — Figure S4 [file CAM4-11-5113-s003.tif]

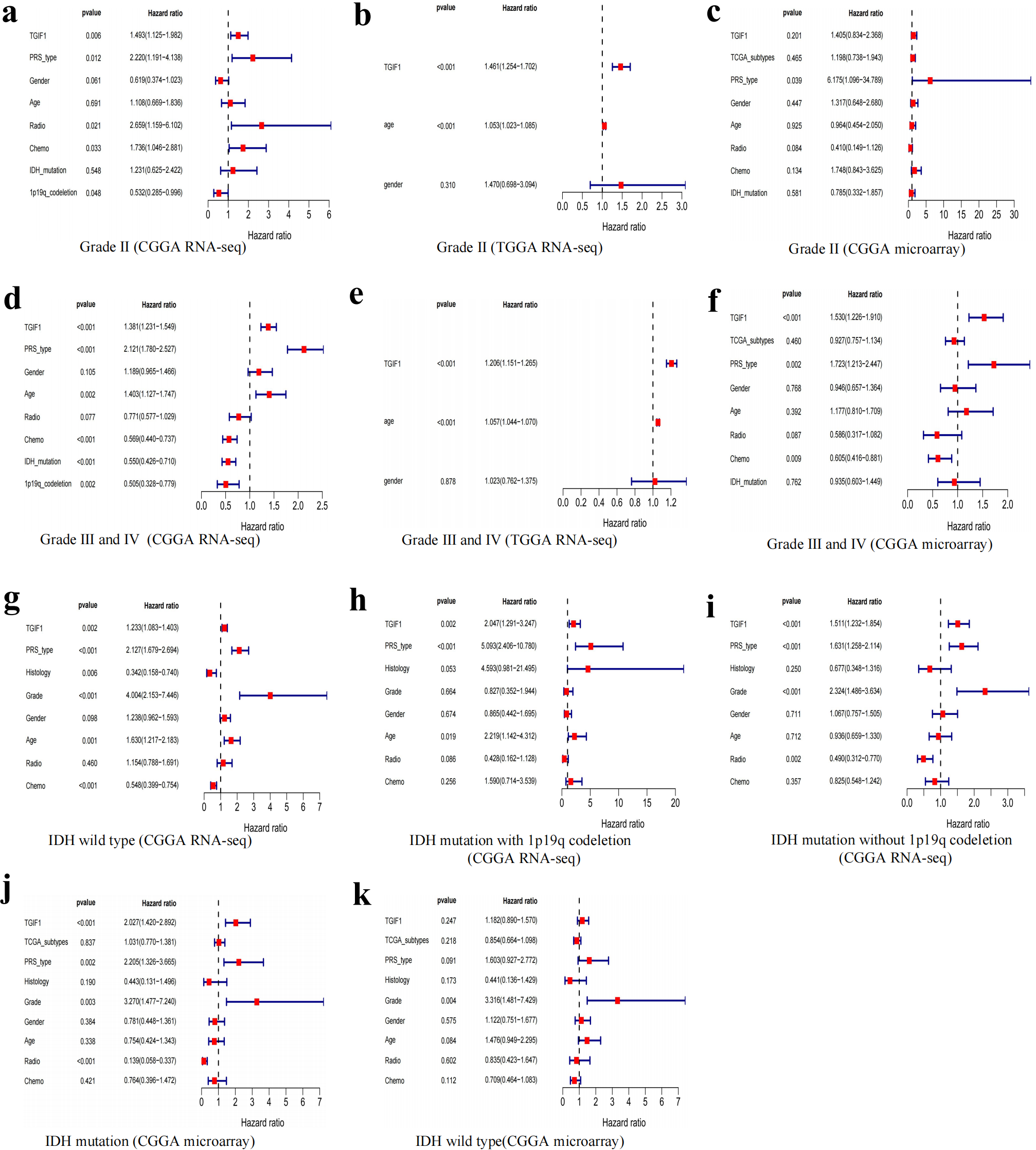

Supplement: Supplementary file 5 — Figure S5 [file CAM4-11-5113-s001.tif]

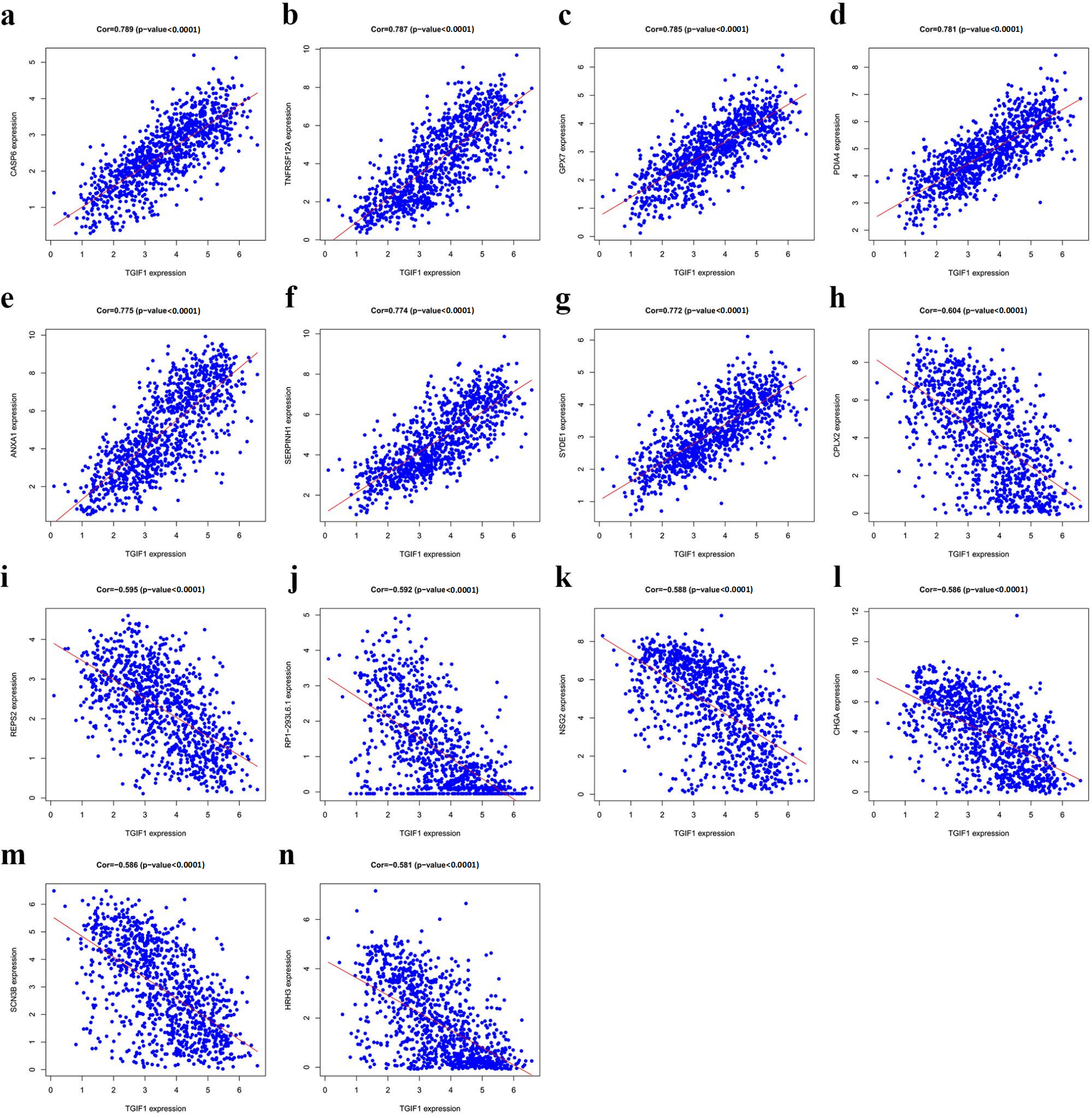

Supplement: Supplementary file 6 — Figure S6 [file CAM4-11-5113-s005.tif]

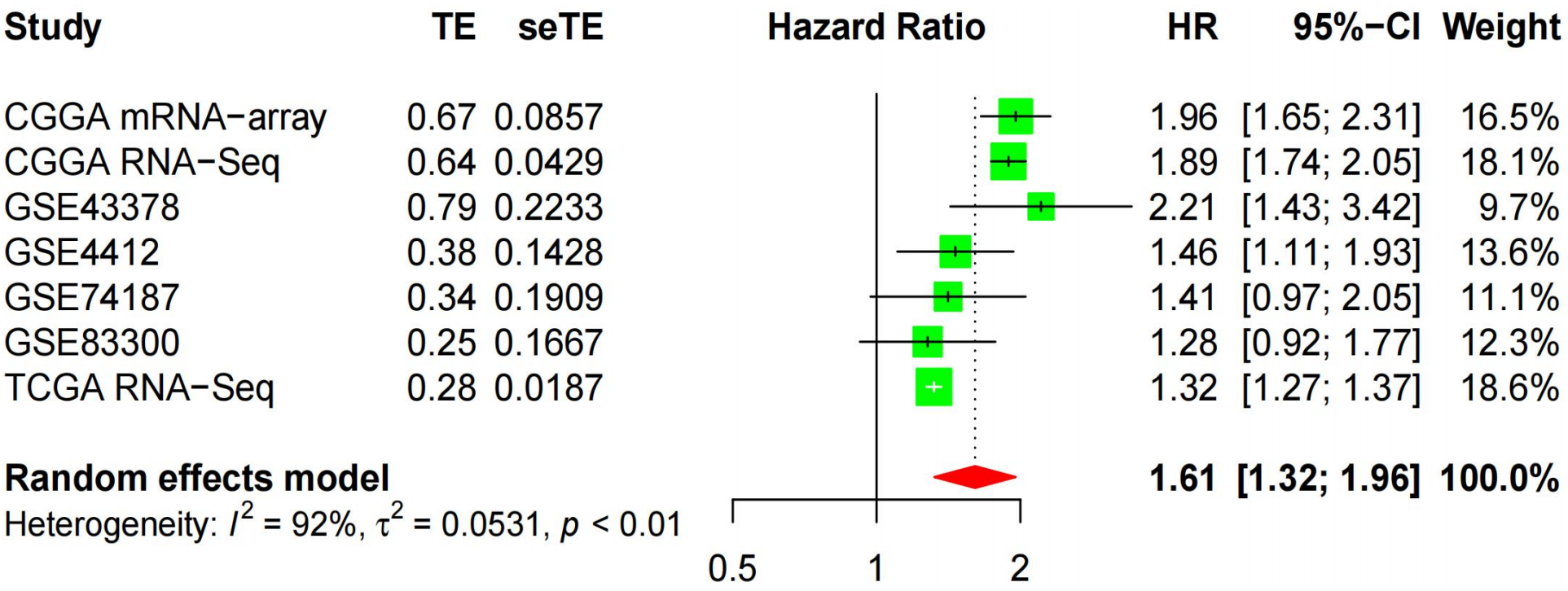

Supplement: Supplementary file 7 — Figure S7 [file CAM4-11-5113-s004.tif]
